# Research Progress on the Multitarget Mechanisms of Terpenoids in the Treatment of Ischemic Stroke

**DOI:** 10.1155/srat/4347247

**Published:** 2026-07-31

**Authors:** Jiao Yang, Zhifeng Wang, Ting Shi, Shiyuan Wu, Pengfen He, Yuxin Han, Daman Tian, Shuangfeng Xu, Junfeng Lan, Yujiang Xi, Pan Pan, Jian Wang

**Affiliations:** ^1^ College of Acupuncture and Massage, Yunnan University of Chinese Medicine, Kunming, China, ynutcm.edu.cn; ^2^ The First Clinical Medical College, Yunnan University of Chinese Medicine, Kunming, China, ynutcm.edu.cn; ^3^ School of Chinese Materia Medica, Yunnan University of Chinese Medicine, Kunming, China, ynutcm.edu.cn; ^4^ School of Nursing, Yunnan University of Chinese Medicine, Kunming, China, ynutcm.edu.cn

**Keywords:** bioavailability, cerebral ischemic stroke, multitarget regulation, neuroprotective mechanisms, terpenoids

## Abstract

Cerebral ischemic stroke (CIS) is an acute cerebrovascular disease associated with high morbidity and disability rates. It is characterized by neurological damage caused by the interruption of cerebral blood flow, often accompanied by sequelae such as motor dysfunction and cognitive decline, which seriously affect the quality of life of patients. Despite continuous advancements in treatment methods, challenges persist, such as time window constraints, limited efficacy, and potential adverse reactions. Therefore, finding more proactive and effective treatment approaches has become an urgent problem to be solved. The mechanism of action of terpenoids is not a simple single‐target inhibition. Essentially, it lies in the systematic remodeling of the postischemic neural microenvironment. The pathophysiological process of CIS involves complex cascades, including energy failure, excitotoxicity, oxidative stress, neuroinflammation, disruption of the blood–brain barrier, multiple cell death pathways, mitochondrial dysfunction, and impairment of nerve repair. Terpenoids can intervene in ischemic stroke through a dual‐mechanism approach of direct neuroprotection and indirect microenvironment regulation by targeting key nodes within the abovementioned pathological network, ultimately achieving the protection and restoration of neural function. The aim of this review was to deepen the understanding of the therapeutic value of terpenoids in stroke; from an integrated perspective, it revealed the scientific essence of the multitarget synergistic effects of natural products, thereby providing a theoretical cornerstone and an innovative direction for the development of next‐generation drugs for treating stroke.

## 1. Introduction

Cerebral ischemic stroke (CIS) is a highly prevalent disease of the central nervous system. It currently ranks as the second leading cause of death worldwide, and its mortality and disability rates continue to increase. According to the World Stroke Report estimates, the global cost of ischemic stroke exceeds $890 billion annually, accounting for 0.66% of global GDP [1]. From 1990 to 2021, the disease burden has significantly increased. Patients with CIS often experience severe sequelae such as hemiplegia, aphasia, and cognitive dysfunction, accompanied by psychological problems such as depression and anxiety. These greatly impair the quality of life of patients and impose a heavy burden on both their families and society. At present, commonly used clinical antiplatelet drugs, statins, and various neuroprotective agents have limited effects on long‐term neurological function improvement and may be accompanied by risks such as bleeding and liver injury [2]. Compared with traditional chemically synthesized drugs, terpenoids are derived from nature and have significant advantages in terms of safety and biocompatibility. They generally exhibit characteristics such as low toxicity and multitarget regulation [3]. They are widely present in foods and herbs and possess various activities such as antibacterial, anti‐inflammatory, antitumor, and neuroprotective properties. Although numerous studies have explored the potential of terpenoids in the treatment of ischemic stroke, their core mechanisms of action and pathway networks remain incompletely elucidated. Therefore, this paper advocates shifting the research perspective from a simple listing of mechanisms to network integration, deepening from neuroprotection to microenvironment remodeling, and systematically elaborating on how terpenoids, through multidimensional interventions, transform the proinflammatory and imbalanced intracerebral microenvironment into an anti‐inflammatory and homeostatic reparative state. This provides a theoretical basis for their clinical application and the development of new drugs.

## 2. Integrated Framework for the Multitarget Intervention of Terpenoids in Ischemic Stroke

**Figure 1:**
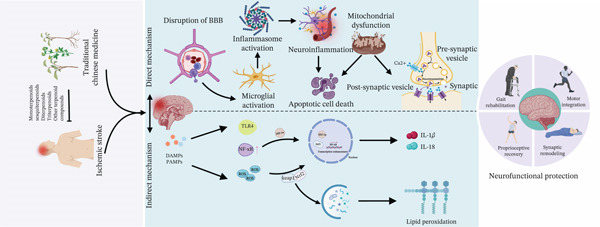
Main neuroprotective mechanisms of terpenoids.

CIS is not merely a simple event of blood vessel blockage. Instead, it is a neural microenvironment tempest triggered by an initial energy crisis. Its pathophysiological process involves multistep cascade reactions, with each step intertwined and mutually causal, creating an out‐of‐control pathological microenvironment. Within minutes of the interruption of cerebral blood flow, the supply of oxygen and glucose is cut off, leading to ATP depletion. The failure of ion pumps causes neuronal depolarization. A large amount of glutamate is released and there is an uptake disorder. Overactivation of NMDA receptors results in Ca^2+^ influx [4, 5]. Subsequently, intracellular calcium overload disrupts mitochondrial function. The electron transport chain leaks, generating excessive reactive oxygen species (ROS). Meanwhile, the collapse of the body′s antioxidant defense system triggers oxidative stress. This not only directly damages biological macromolecules but also activates downstream inflammatory and cell death pathways, amplifying the damage effect [6]. Subsequently, damage‐associated molecular patterns (DAMPs) released by oxidative stress activate glial cells, which release proinflammatory cytokines and chemokines to recruit peripheral immune cell infiltration, forming an inflammatory storm. Inflammatory cytokines and matrix metalloproteinases (MMPs) degrade tight‐junction proteins, disrupting the integrity of the blood–brain barrier, and exacerbating the vicious cycle of cerebral edema and inflammation [7]. Ultimately, under multiple blows, nerve cells initiate various programmed cell death methods such as apoptosis, pyroptosis, and ferroptosis, which promote each other, leading to neuronal loss and neurological deficits. The CIS pathological network starts with energy failure and excitotoxicity, is amplified through oxidative stress and mitochondrial dysfunction, triggers the collapse of the microenvironment, and finally ends with multimode cell death, forming a positive‐feedback deteriorating cycle. This complex network provides the core basis for the multitarget intervention of terpenoids. As shown in Figure 1, terpenoids can achieve the protection and restoration of neural function through a dual‐mechanism approach of direct neuroprotection and indirect microenvironment regulation.

Terpenoids exert their effects in ischemic stroke through a dual mechanism involving direct neuroprotection and indirect microenvironment regulation. The direct mechanism involves the poststroke pathological cascade, blocking neural damage at the cellular and molecular levels by influencing the integrity of the blood–brain barrier, inhibiting inflammasome activation and the spread of neuroinflammation, mitigating mitochondrial dysfunction, and protecting against synaptic damage and neuronal apoptosis. In the indirect mechanism, terpenoids activate the TLR4/NF‐*κ*B and Nrf2 signaling pathways via DAMPs and pathogen‐associated molecular patterns (PAMPs), alleviate oxidative stress, inhibit the release of the inflammatory factors interleukin (IL)‐1*β* and IL‐18, and remodel the neural microenvironment. Ultimately, these actions promote the protection and recovery of neurological function and illustrate the unique advantages and scientific rationale of traditional Chinese medicine (TCM) in achieving synergistic effects through multicomponent and multitarget intervention. (This figure was created by the authors).

## 3. Neuroprotective Effects of Terpenoids and the Underlying Mechanisms

Terpenoids have shown great application potential for the treatment of ischemic stroke. The ultimate goal of neuroprotection is the restoration of neurological function. The pathophysiological improvements mediated by terpenoids, such as reduced infarct volume and suppressed neuroinflammation, must translate into tangible behavioral recovery. Several human, animal, and in vitro studies have shown the efficacy of plant‐ and microorganism‐derived terpenoids in providing neuroprotection in ischemic stroke. Owing to their diverse pharmacological and biochemical properties, terpenoids can significantly contribute to counteracting the progression of ischemic stroke. Therefore, understanding the complementary advantages and disadvantages of each compound is of great practical significance for choosing the direction of clinical translation and allocating research resources. Based on systematic reviews, meta‐analyses, randomized controlled trials, and preclinical studies published in multiple databases and high‐impact journals, the differences between terpenoids and neural repair strategies were compared, as shown in Table 1.

**Table 1:** Terpenoids in clinical trials and basic experiments in ischemic stroke.

	Constituent	The targeted pathway of the action	Principal research results	Research gaps	References
Monoterpenoids	Catalpol	The VEGF‐A/KDR pathway, the SDF‐1*α*/CXCR4 pathway, the JAK2/STAT3 pathway, and the NLRP3 inflammasome	To promote angiogenesis, the proliferation and differentiation of neural stem cells, as well as their directed migration; to inhibit the NLRP3 inflammasome and oxidative stress; and to increase cerebral blood flow.	The specific molecular navigation mechanisms underlying the promotion of the directed migration of neural stem cells to the ischemic cortex, as well as the regulatory discrepancies among neural stem cells from diverse origins, remain undefined.	[8–12]
	Paeoniflorin	The Keap1‐Nrf2 pathway, the AMPK‐ULK1‐TFEB pathway, and the NLRP3 inflammasome	Augment the activity of antioxidant enzymes, suppress the release of proinflammatory factors, and initiate the autophagy pathway to eliminate inflammatory damage.	The upstream signaling network responsible for activating the AMPK‐ULK1‐TFEB pathway to augment autophagic flux has not been characterized. Moreover, direct evidence regarding the causal relationship between enhanced autophagy and the inhibition of neuroinflammation is lacking.	[13–15]
	Borneol	Permeabilize the blood–brain barrier	Synergistically disrupt the blood–brain barrier in conjunction with drugs, facilitate neurogenesis and glial cell repair, and preserve the integrity of the barrier. When combined with edaravone, the proportion of patients with mRS ≤ 1 significantly increases after 90 days.	There is a lack of systematic research on the molecular mechanisms underlying the transient opening of the blood–brain barrier, as well as the optimal ratios and time windows of its synergistic interactions with various drugs.	[16, 17]

Sesquiterpenoids	Artemisinin	The TXNIP/NLRP3/caspase‐1 signaling axis, cGAS‐STING pathway, and mitochondrial complex I	Inhibition of mitochondrial complex I reduces the production of ROS, blocks the inflammatory signaling axis, activates lysosomal function to suppress the cGAS‐STING pathway, thereby alleviating neuroinflammation and oxidative stress‐induced damage.	The molecular binding sites for the inhibition of mitochondrial complex I and their potential impacts on other complexes have yet to be determined. Additionally, no molecular link mediating the inhibition of the cGAS‐STING pathway via lysosomes has been identified.	[18, 19]
	Caryophyllene	The TLR4/NF‐*κ*B signaling pathway, the RAGE/NF‐*κ*B signaling pathway, and the Nrf2/HO‐1 signaling pathway	Downregulating HMGB1 blocks the binding of TLR4/RAGE, inhibits the NF‐*κ*B pathway, thereby alleviating inflammation. Activating the Nrf2/HO‐1 pathway inhibits ferroptosis, reduces brain injury, and facilitates neural recovery.	The temporal relationship between the regulation of Nrf2 nuclear translocation and the expression of ferroptosis‐related proteins, as well as the cell‐specific mechanisms, remain undefined.	[20, 21]

Diterpenoids	Andrographolide	The Nrf2 signaling pathway	Inhibit the activation of astrocytes, promote the survival of neurons, reduce the levels of proinflammatory factors, and activate the Nrf2 pathway to safeguard the integrity of vascular endothelium.	The downstream effector molecules involved in protecting the vascular endothelium via the Nrf2 pathway, as well as its nonantioxidant functions, remain poorly understood.	[22–24]
	Triptolide	The BDNF‐Akt signaling pathway, the Wnt/*β*‐catenin signaling pathway, and the Fractalkine/CX3CR1 signaling pathway	Activating the relevant pathways, triggering autophagic flux, promoting synaptic plasticity, and inhibiting the M1 polarization of microglia to mitigate neuroinflammation.	The regulatory mechanisms of BDNF secretion patterns and the differences in their effects on different types of synapses have not been elucidated.	[25–27]
	Ginkgolide B	The CCT/TRiC‐SK1 axis, the PINK1‐Parkin pathway, and the NLRP3 inflammasome	Activation of the CCT/TRiC‐SK1 axis promotes angiogenesis, induces mitophagy, and inhibits the NLRP3 inflammasome.	The molecular interactions underlying angiogenesis promoted by the CCT/TRiC‐SK1 axis, as well as the signaling crosstalk between mitophagy and the inhibition of the NLRP3 inflammasome, remain to be elucidated.	[28–31]

Triterpenoid compounds	Ginsenoside	The HIF‐1*α*/VEGF signaling pathway and mitochondrial complex I	Suppress the excessive activation of glial cells, mitigate mitochondrial damage, and activate the HIF‐1*α*/VEGF pathway to promote neovascularization.	The molecular mechanisms underlying the inhibition of mitochondrial complex I in astrocytes and the transport mechanisms of mitochondrial transfer to neurons remain elusive.	[32–35]
	Notoginsenoside R1	The Nrf2/HO‐1 pathway, the NF‐*κ*B signaling pathway, the NLRP3 inflammasome, VEGF, and the Ang‐1/Tie2 signaling pathway	Activate the Nrf2/HO‐1 pathway to counteract oxidative damage, inhibit NF‐*κ*B and NLRP3 to mitigate inflammation, promote neovascularization, and reduce thrombosis.	The association between the regulation of gut microbiota and neuroprotection, along with the bioactivity of microbiota‐derived metabolites, remains unvalidated. Additionally, the alterations in its role within models of comorbid metabolic diseases remain unknown.	[36, 37]

Other terpenoids	Astaxanthin	Exhibit antioxidant effects and inhibit apoptosis	Significantly mitigate the volume of cerebral infarction, ameliorate neurological deficits, decrease oxidative stress, inhibit apoptosis, and modulate the expression of inflammatory genes.	The bioavailability of natural astaxanthin is comparatively low. At present, the evaluation of its bioavailability predominantly depends on in vitro digestion models, while in vivo research focusing on absorption and metabolism remains scarce.	[37–39]

### 3.1. Monoterpenoids

Monoterpenoids are characterized by the presence of two isoprene units and small molecular masses. Moreover, they have good blood–brain barrier permeating ability and exhibit favorable neuroprotective effects in the treatment of CIS [40]. They can play a crucial role at the forefront and early stages of the pathological network, clearing obstacles and creating conditions for subsequent in‐depth repair.

Catalpol is an iridoid glycoside and the main active ingredient in TCMs such as *Rehmannia glutinosa* [41]. In the CIS model, catalpol exerts its core function by promoting nerve repair. Studies have shown [8, 9] that catalpol can increase the number of BrdU+/Nestin–positive cells and neural stem cells in the subventricular zone (SVZ), induce their directional migration to the ischemic cortex, and simultaneously enhance the expression of angiogenesis‐related factors to promote the establishment of collateral circulation. This structural restoration in the peri‐infarct region is functionally significant, as it correlates with the recovery of sensorimotor functions. Experimental data often demonstrate this as a reduction in neurological severity scores and improved performance in motor coordination tests. At the levels of inflammation and oxidative stress, catalpol scavenges mitochondrial ROS, which targets and inhibits the assembly of the NLRP3 inflammasome. Since mitochondrial dysfunction is a direct consequence of downstream calcium overload caused by excitotoxicity, the protective effect of catalpol on mitochondrial function can indirectly inhibit glutamate‐mediated excitotoxic damage [10], promotes the polarization of microglia towards the anti‐inflammatory M2 phenotype, effectively alleviates neuroinflammatory damage, promotes their migration to the ischemic cortex, and has a significant angiogenesis‐promoting effect [11]. In addition, catalpol can also increase cerebral blood flow, activate the signal transducer and activator of transcription 3 (STAT3) signaling pathway, and restore the binding activity between STAT3 and vascular endothelial growth factor (VEGF) [12], further synergistically promoting neural repair and vascular remodeling.

Paeoniflorin, a monoterpene glycoside, is a key active ingredient in TCM materials such as paeonia rubra. Its unique “cage‐like” pinane skeleton structure endows it with diverse biological activities [42]. It exhibits the characteristic of multimechanism synergy in counteracting the neuroinflammatory damage caused by ischemic stroke. Experimental studies [13] have shown that paeoniflorin can significantly reduce infarct volume and improve neurological function scores. On the one hand, it enhances the activity of superoxide dismutase (SOD) and glutathione peroxidase (GPX), accelerates free radical scavenging, and alleviates oxidative stress damage. On the other hand [14], it blocks the inflammatory cascade via the activation of the Keap1/Nrf2 signaling pathway and achieves neuroprotection [15]; specifically, paeoniflorin inhibits the formation of the NLRP3 inflammasome. Moreover, by targeting different nodes such as AMPK, ULK1, and TFEB, it jointly promotes the generation of LC3‐II and the degradation of p62, enhances autophagic flux, combats the misfolding of proteins caused by endoplasmic reticulum stress, and alleviates endoplasmic reticulum stress‐induced neuronal apoptosis. Consequently, this more thoroughly clearing inflammatory stimuli and damaged components, reducing the release of inflammatory factors such as IL‐6 and IL‐1*β*, to achieve a more profound inhibition of neuroinflammation.

Borneol is a monoterpenoid derived from plants of the Dipterocarpaceae family and can also be synthesized via the reduction of camphor. The effects of borneol mainly rely on its strong lipophilicity, which induces the transient opening of the blood–brain barrier and significantly improves the efficiency of the intracerebral delivery of neuroprotective drugs [43, 44]. An experimental study on the combined application of borneol and *Ligusticum chuanxiong* [16] showed that their combined use can synergistically promote neurogenesis as well as the transformation of astrocytes to the A2 phenotype. Meanwhile, by upregulating the expression of tight junction proteins, they synergistically preserve the integrity of the blood–brain barrier, thereby contributing to the protection and repair of the cerebral microenvironment. A Phase III randomized, double‐blind, controlled trial conducted in 48 hospitals in China showed [17] that for patients with acute ischemic stroke within 48 h of onset, the treatment regimen of edaravone combined with d‐borneol significantly increased the proportion of patients with a modified Rankin Scale (mRS) score ≤ 1 after 90 days of treatment, compared with edaravone alone. The efficacy was particularly prominent in the female patient group, fully demonstrating the synergistic potential of borneol in new drug combinations for ischemic stroke. The improvement in mRS scores, particularly the increased proportion of patients achieving mRS ≤ 1, indicates a significant enhancement in activities of daily living, which heavily relies on motor function recovery.

Monoterpenoids, with their small molecular weight and good blood–brain barrier permeability, mainly act on the front line of the pathological network. They are characterized by clearing early obstacles and paving the way for subsequent repair. Their core mechanism lies in the simultaneous or sequential regulation of multiple early processes such as neurogenesis, oxidative stress, and neuroinflammation, demonstrating great potential for intervention in the acute phase.

### 3.2. Sesquiterpenoids

Sesquiterpenoids are C15‐terpenoid compounds composed of three isoprene units and are mainly present in the essential oils of fresh plant materials. They represent the most diverse class within terpenoids [45]. These compounds can dynamically coordinate the intertwined pathological processes of neuroinflammation and ferroptosis, highlighting their unique advantage of multitarget regulation.

Artemisinin, an active sesquiterpene lactone compound with a peroxide bridge structure extracted from the Chinese medicinal herb *Artemisia annua*, is among the most extensively studied molecules in current sesquiterpenoid research. Its liposolubility and small molecular mass enable it to effectively cross the blood–brain barrier [46]. Recent studies have demonstrated that artemisinin and its derivatives exhibit significant neuroprotective activity in the treatment of ischemic stroke. For instance, artemisinin can reduce the production of ROS by inhibiting mitochondrial complex I, thereby blocking the TXNIP/NLRP3/caspase‐1 signaling axis and suppressing pyroptosis and inflammatory responses [18]. In addition, Wei et al. [19] found that artemisinin activates lysosomal function by upregulating the expression of cathepsin B/D and lysosome‐associated membrane protein 2 (LAMP2), promoting intracellular DNA degradation. This leads to the inhibition of the cGAS‐STING signaling pathway, and, ultimately, a decline in the release of key inflammatory factors such as IL‐1*β* and TNF‐*α*, thereby effectively alleviating neuroinflammation and oxidative stress damage.

Caryophyllene is a naturally occurring bicyclic sesquiterpene compound found in the essential oils of plants such as clove and cinnamon [47]. In the context of ischemic stroke, studies have indicated that caryophyllene downregulates the expression of high‐mobility group box 1 (HMGB1), a key DAMP, and blocks its binding to TLR4 to inhibit the activation of the downstream NF‐*κ*B signaling pathway [20]. Hu et al. [21] demonstrated that caryophyllene can effectively activate the nuclear factor erythroid 2‐related factor 2/heme oxygenase‐1 (Nrf2/HO‐1) signaling pathway, promote Nrf2 nuclear translocation, upregulate the expression of HO‐1 and GPX4, and inhibit that of acyl‐CoA synthetase long‐chain family member 4 (ACSL4) and cyclooxygenase‐2 (COX2). This reduces iron ion accumulation and lipid peroxidation, inhibits ferroptosis in nerve cells, and promotes neurological function recovery.

Sesquiterpenoids are notably characterized by dynamically coordinating the two intertwined pathological processes of neuroinflammation and ferroptosis, demonstrating the unique advantage of multitarget regulation. They effectively block cell death driven by oxidative stress and inflammation by inhibiting inflammatory pathways and activating the antioxidant defense system, thus playing a crucial “braking” role in the intermediate stage of the pathological network of CIS.

### 3.3. Diterpenoids

Diterpenoids are a class of natural terpenoids polymerized from four isoprene units, and their derivatives form diverse molecular skeletons through structural modifications [48]. In the middle and late stages of the pathological process, its focus of action clearly shifts to the dynamic intervention and repair of mitochondrial function and synaptic structure.

Andrographolide, a terpenoid compound extracted from the traditional Chinese medicinal herb *Andrographis paniculata* [49]. Studies [22, 23] have shown that it significantly downregulates the expression of glial fibrillary acidic protein (GFAP), a marker of activated astrocytes, while upregulating that of neuronal nuclear antigen (NeuN), suggesting that it inhibits neuroinflammation and promotes neuronal survival. Notably, andrographolide also possesses important vascular‐protective functions.

Triptolide, a cyclic epoxy diterpene lactone compound, is the main active component of the *Tripterygium wilfordii* plant [24]. In a mouse model of ischemic stroke, pretreatment with triptolide significantly improved neurological behavioral deficits [25]. In‐depth mechanistic studies have shown that triptolide upregulates the levels of brain‐derived neurotrophic factor (BDNF) in brain tissue, enhances AKT (protein kinase B) phosphorylation and the activity of extracellular signal‐regulated kinase 1/2 (ERK1/2), significantly elevates the expression levels of synapse‐related proteins, and increases dendritic spine density. Given that synaptic plasticity is the cellular basis of learning and memory, these findings suggest that triptolide has the potential to ameliorate poststroke cognitive impairment (PSCI). This is further supported by behavioral tests such as the Morris water maze (MWM), where treated subjects exhibit shorter escape latencies, indicating improved spatial memory [26].

Ginkgolide B is a diterpene lactone compound extracted from Ginkgo biloba leaves. Recent studies have shown that ginkgolide B specifically binds to and inhibits the activity of creatine kinase B (CKB), leading to the upregulation of the expression of the beta subunit of the chaperonin complex chaperonin containing TCP‐1 (CCT‐*β*) [27], which, in turn, promotes the proper folding and maturation of sphingosine kinase 1 (SK1). By activating this CCT/TRiC‐SK1 signaling axis, ginkgolide B ultimately exerts a proangiogenic effect, thus supporting neural repair. Liang et al. [28] demonstrated that ginkgolide B induces mitophagy by activating PINK1‐Parkin, remove damaged mitochondria. The endoplasmic reticulum and mitochondria are in close contact at a structure called the mitochondria‐associated endoplasmic reticulum membrane (MAMs), which regulates calcium homeostasis. The autophagy‐inducing effect of ginkgolide B on mitochondria can restore the calcium balance between the endoplasmic reticulum and mitochondria, thereby alleviating endoplasmic reticulum stress caused by ischemic stress, and inhibit the release of NLRP3 inflammasome and IL‐1*β*, thereby significantly blocking neuroinflammatory cascade‐mediated damage. Importantly, the neuroprotective effect of ginkgolide B has been supported by clinical studies [29]. Multiple multicenter, randomized, double‐blind clinical studies have shown [30, 31] that the proportion of patients with good mRS results at 90 days in the group treated with ginkgolide B is significantly higher than that in the control group.

The focus of the action of diterpenoids clearly shifts to the dynamic intervention and repair of mitochondrial function and synaptic structure. They can not only protect mitochondria from damage and remove damaged organelles, but also promote the expression of neurotrophic factors and synaptic plasticity. In the middle and late stages of the pathological process, diterpenoids directly maintain and support the survival and information‐transfer functions of neurons.

### 3.4. Triterpenoids

Triterpenoids are natural C30‐terpenoid compounds formed by the linkage of six isoprene units (C_5_H_8_) in a head‐to‐head or head‐to‐tail arrangement. They have a diversity of molecular skeletons, ranging from linear chains to monocyclic, bicyclic, and pentacyclic structures [50, 51]. This type of compound continuously promotes the integrative repair of the neurovascular unit throughout the pathological process by dynamically regulating angiogenesis and glial cell functions [52, 53].

Ginsenosides, including the triterpene‐type Rg1 and the diol‐type Rb1, are the main active components responsible for the pharmacological effects of ginseng. In a mouse model of ischemic hypoxic brain injury [32], ginsenoside exerted significant anti‐inflammatory, antiapoptotic, and neuroprotective effects by inhibiting excessive microglia activation and mitigating mitochondrial damage in astrocytes. Furthermore, ginsenosides can promote the proliferation and migration of brain microvascular BMECs, activate the HIF‐1*α*/VEGF signaling pathway, promote the differentiation of mesenchymal stem cells (MSC), and enhance angiogenesis and microvascular density, while simultaneously stimulating the proliferation of neurons and astrocytes, ultimately reducing the volume of cerebral infarction and improving neurobehavioral function [33]. Mechanistically, ginsenosides create a supportive microenvironment by inhibiting excessive activation of astrocytes and promoting angiogenesis, which sustains the survival of neurons. Ni et al. [34] demonstrated that ginsenoside Rb1 reversibly inhibits the activity of mitochondrial complex I in astrocytes, significantly reducing the ROS burst triggered by reverse electron transport both in vivo and in vitro. This effect inhibits the excessive activation of astrocytes, maintains their normal metabolic support functions, thereby significantly ameliorating ischemic brain injury. In a randomized, double‐blind, placebo‐controlled clinical trial [35], it was found that ginsenoside Rd significantly improved the mRS scores of patients with nonlacunar stroke at 3 months, alleviated the degree of their disability at 90 days, and promoted the recovery of neurological function.

Saponin R1 is the main active component isolated from *Panax notoginseng*, and is uniquely found in its roots. It has been shown that *P. notoginseng* saponin R1 can activate the Nrf2/HO‐1 pathway, effectively inhibiting oxidative stress‐induced damage, and also significantly alleviates neuroinflammatory responses by blocking NF‐*κ*B signaling and inhibiting NLRP3 inflammasome activation [54]. Regarding its effect on cerebral blood flow [36], *P. notoginseng* saponin promotes angiogenesis through the VEGF and Ang‐1/Tie2 signaling pathways, and reduces thrombosis by inhibiting platelet‐activating factor (PAF). Clinical studies have shown that Xuesaitong injection, which contains total saponins from *P. notoginseng*, significantly improves patients′ NIHSS scores and reduces the stroke recurrence rate.

Triterpenoids promote the integrated repair of the neurovascular unit throughout the pathological process by dynamically regulating angiogenesis and glial cell functions. They can simultaneously activate prosurvival signaling pathways and angiogenesis pathways, and modulate the states of microglia and astrocytes. Thus, they systematically reshape the postinjury microenvironment, providing a structural basis for long‐term neurological function recovery.

### 3.5. Other Terpenoids

The mechanisms behind the pathology of ischemic stroke are complex. Apart from monoterpenoids, sesquiterpenoids, diterpenoids, and triterpenoids, other terpenoid compounds also exhibit unique multitarget therapeutic potential. Astaxanthin, a naturally occurring and bioactive tetraterpenoid, possesses potent antioxidant properties, effectively scavenging free radicals and inhibiting lipid peroxidation [37]. Research findings indicate that astaxanthin markedly decreases infarct volume in MCAO model rats and facilitates the improvement of neurological deficits [38]. It can also restore the total oxidative status (TOS) and the level of caspase‐3 to normal, enhance the activity of the antioxidant enzyme GPX, and promote the expression of the NF‐*κ*B gene. Furthermore, astaxanthin downregulates the expression of the proapoptotic gene *BAX* and restores the expression of BCL‐2 to normal levels. Astaxanthin was also shown [39] to significantly mitigate ischemia‐induced oxidative DNA damage and lipid peroxidation in pyramidal cells within the CA1–CA3 regions, thereby alleviating oxidative stress‐induced injury.

### 3.6. Comprehensive Synergistic Patterns of Terpenoid Compounds

Terpenoid compounds demonstrate unique advantages in addressing ischemic stroke. The core lies in breaking through the limitations of the effects of a single component. Through the precise coordination of multiple subclass components in time and space, a systematic network regulation mechanism is formed. This synergy is not a simple accumulation of effects but a qualitative leap achieved by reconstructing the pathological process and reversing the vicious cycle. In the acute phase, borneol, a monoterpenoid, can, by virtue of its strong lipophilicity, induce the transient opening of the blood–brain barrier, creating a crucial window for subsequent drug delivery [43]. The opening of this channel enables ginkgolide B, a diterpenoid, to enter the brain efficiently. Subsequently, it activates the PINK1‐Parkin pathway, clears dysfunctional mitochondria, and effectively suppresses the activation of the NLRP3 inflammasome, completing the sequential process from opening the channel to core intervention [55]. At the same time, caryophyllene, a sesquiterpenoid, inhibits ferroptosis by activating the Nrf2/HO‐1 pathway. It effectively clears lipid peroxides and inflammatory factors in the microenvironment. This clears the microenvironmental obstacles for ginsenosides, triterpenoids, to promote angiogenesis through the HIF‐1*α*/VEGF pathway, laying a solid foundation for nerve repair [20]. During the repair stage, triptolide, a diterpenoid, focuses on enhancing synaptic plasticity and neuron survival. Meanwhile, notoginsenoside R1, a triterpenoid, is responsible for reconstructing the microcirculation through the VEGF and Ang‐1/Tie2 signaling pathways. Starting from the cellular and systemic levels, respectively, the two jointly complete the systematic nerve function repair project [56, 57]. This integrated strategy featuring sequential action in time and space, cross‐fire effects, and functional complementarity profoundly interprets the scientific connotation of the multitarget synergy of natural products. It offers a novel treatment paradigm for tackling complex brain diseases.

### 3.7. Clinical Status and Evidence

Although a large number of basic studies have confirmed the neuroprotective potential of terpenoids, the process of their clinical translation is still in the early stages. Currently, only a few compounds have entered clinical trials. For example, a Phase III randomized, double‐blind, controlled trial conducted in 48 hospitals in China showed that, for patients with acute CIS within 48 h of onset, the treatment regimen of edaravone combined with d‐borneol could significantly increase the proportion of patients with a mRS score ≤ 1 after 90 days, compared with the use of edaravone alone [58, 59]. This effect was particularly prominent in female patients. This fully demonstrates the synergistic potential of borneol in new drug combinations. In addition, the neuroprotective effect of ginkgolide B has been supported by clinical studies. Multiple multicenter, randomized, double‐blind clinical studies have shown that [60] the proportion of patients in the ginkgolide B treatment group achieving a favorable mRS outcome after 90 days was significantly higher than that in the control group. In a randomized, double‐blind, placebo‐controlled clinical trial [35, 61], ginsenoside Rd was found to significantly improve the mRS score of patients with nonlacunar stroke at 3 months, reduce disability at 90 days, and promote neurological function recovery. Moreover [62], Xuesaitong injection, which contains total notoginsenosides, has also been proven in clinical studies to significantly improve patients′ NIHSS scores and reduce the stroke recurrence rate.

However, the clinical data of most terpenoids remain extremely limited, lacking large‐scale, multicenter validation. The sample sizes of existing studies are generally small, and they mainly focus on short‐term neurological function improvement, with insufficient assessment of long‐term endpoints such as cognitive function and quality of life. One of the main bottlenecks in clinical translation is the low bioavailability of terpenoids, which is attributed to their unfavorable physicochemical properties (e.g., low water solubility and poor stability) and the limitations of physiological barriers. Therefore, future research needs to incorporate novel delivery systems (e.g., mitochondria‐targeted liposomes) to overcome these obstacles.

## 4. Remote Regulation via the Gut–Brain Axis: A New Gut‐Based Pathway for Terpenoid Compounds to Reshape the Poststroke Neural Microenvironment

Terpenoid compounds exhibit multitarget and multipathway composite effects in the neuroprotection of ischemic stroke. The onset of ischemic stroke is closely related to systemic metabolic disorders, and the gut microbiota is an important link in regulating blood lipid, blood pressure, and atherosclerosis. During the pathological process of ischemic stroke, ischemia and hypoxia not only severely affect the brain tissue but also, through the systemic stress response, activate the hypothalamic–pituitary–adrenal axis (HPA axis) and stimulate the sympathetic nerves. Together, these lead to gut microbiota dysbiosis and alter the balance of the gut microecology. This change, in turn, acts on the central nervous system via the “gut–brain axis,” creating a vicious cycle that exacerbates brain injury [63, 64]. Combining the direct neuroprotective effects of terpenoid compounds with the regulatory role of the brain–gut axis can form a “two‐way” intervention strategy. Terpenoids directly protect the ischemic brain tissue by inhibiting platelet aggregation, antioxidation, and antiapoptosis [65]. In addition, they can promote the growth of beneficial bacteria, reduce the abundance of pathogenic bacteria, thereby reducing the systemic release of enterogenic inflammatory mediators, and alleviating poststroke immune disorders and complications [66, 67]. Therefore, in the treatment of ischemic stroke, terpenoid compounds are not only traditional neuroprotectants but may also become key molecules for regulating the brain–gut axis, overall metabolism, and immune balance. This provides a theoretical basis and experimental support for multitarget comprehensive treatment.

## 5. Challenges and Transformation Strategies in Clinical Translation

Terpenoids are a class of compounds derived from natural products. Due to their extensive biological activities and multitarget antineuroinflammatory mechanisms, they exhibit great potential in the treatment of CIS. Terpenoids can exert anti‐inflammatory effects not only through traditional means—such as inhibiting the apoptosis of nerve cells and the release of various proinflammatory factors—but also by enhancing the immune sensitivity of autoimmune cells or inducing microglia polarization to resist neuroinflammation. Moreover, they have shown significant therapeutic effects in CIS injury models.

However, despite the positive results in laboratory research, the clinical translation of terpenoids still faces numerous challenges. The structures of many terpenoids in the clinical trial stage differ significantly from those in the previous tests, and there is often a lack of suitable clinical models. The complex structures of terpenoids limit their large‐scale synthesis, and such complex structures often make it difficult to study the relationship between drug activity and structure. Terpenoids have poor targeting properties, which may kill normal cells at high concentrations, and the poor efficacy caused by low bioavailability is a key issue restricting their clinical application [68–70].

Next, we summarize possible solutions for improving the bioavailability of terpenoids, including the application of mitochondria‐targeted delivery systems, lipid nanocarrier technology, and enzyme and metabolic engineering technologies.

Mitochondria‐targeted delivery systems overcome the limitations of traditional drug delivery through a threefold mechanism, involving electrochemical gradient‐driven transport, ligand–receptor recognition, and stimuli‐responsive drug release. This approach can effectively improve terpenoid enrichment efficiency, bioavailability, and solubility [71, 72]. The conjugation of artesunate and rhein via linker chains of varying lengths allows for precise targeting and enhances cellular permeability, heme‐binding capacity, and iron ion affinity. By controlling the length of the linker chains, this system enables the precise regulation of drug conformation while simultaneously optimizing cellular uptake and target affinity. This approach also overcomes the delivery bottleneck in the application of artesunate, leading to improved drug stability and bioavailability [73]. Moreover, this strategy shows promise for significantly improving the solubility and bioavailability of terpenoids. Pharmacokinetic data indicate that paclitaxel—a terpenoid compound—delivered via mitochondria‐targeted liposomes (TPP‐PEG‐PE conjugates) exhibits enhanced cytotoxicity and antitumor efficacy compared with free paclitaxel or delivery via unmodified conventional liposomes [74, 75]. TPP‐PEG‐PE can be used as a targeting ligand in the preparation of nontoxic, mitochondria‐targeted drug delivery systems. Over recent years, mitochondria‐targeted drug delivery has emerged as a promising drug carrier strategy, offering enhanced drug stability, improved bioavailability, and sustained‐release properties [76]. In mouse models, this preparation significantly improves the in vivo pharmacokinetic parameters of paclitaxel, such as its blood circulation half‐life and area under the drug‐time curve (AUC), thereby prolonging its retention time in the body and reducing its clearance rate [77].

In addition, enzyme and metabolic engineering technologies play a key role in optimizing terpenoid synthesis, regulation, and metabolism. Structural biology has enabled a detailed analysis of key enzymes such as terpene synthases and cytochrome P450 oxidases. Rational design and directed evolution have been applied to modify their activity, stability, and selectivity, significantly improving the yield and specificity of target products [78–80]. Furthermore, through the systematic reconstruction and optimization of terpenoid synthesis pathways, metabolic flux can be balanced using methods such as promoter engineering, RBS optimization, and regulation of gene copy numbers, thereby increasing the content of rare terpenoids in medicinal plants such as *A. annua* and *Taxus* spp. [81, 82]. Enzyme and metabolic engineering have become a powerful driving force for promoting efficient and green manufacturing of terpenoids and the discovery of novel drugs. These two fields complement each other, providing strategies and insights for the efficient production of terpenoids and microbial synthesis of other natural products.

## 6. Conclusions and Prospects

The core value of this review lies in elevating the research perspective of terpenoids in treating CIS from the traditional single‐target inhibition to an integrated framework of systematic remodeling of the neural microenvironment. We found that terpenoids can systematically intervene in the CIS pathological network through a coordinated pattern of spatiotemporal sequencing and functional complementarity. For example, in the acute phase, monoterpenoid borneol can open the blood–brain barrier, creating conditions for the diterpenoid ginkgolide B to enter the brain and clear damaged mitochondria. Meanwhile, the sesquiterpenoid caryophyllene inhibits ferroptosis, removing obstacles for the triterpenoid ginsenoside to promote angiogenesis. Eventually, diterpenoids and triterpenoids collaborate to complete neural repair. This cross‐ and mutual‐assistance integrated strategy is the core innovation that distinguishes this paper from previous studies that merely list mechanisms. In addition, this paper prospectively proposes the gut–brain axis as a new pathway for terpenoids to remotely regulate the neural microenvironment, emphasizing its bidirectional and systematic regulatory role.

Notwithstanding, there are still significant limitations in this field. Firstly, the depth and precision of key molecular mechanisms are insufficient. For instance, the mechanism by which catalpol promotes the migration of neural stem cells and the optimal time window for borneol to open the blood–brain barrier remain to be further explored. Secondly, clinical translation faces major challenges such as low bioavailability and poor blood–brain barrier permeability. Although new technologies like the mitochondrial‐targeted delivery system show promise, their safety and efficacy still need to be systematically evaluated. Finally, most of the conclusions are derived from animal models, and there is an urgent need for more large‐scale clinical trials to verify their efficacy and safety in humans. Future research should focus on filling these mechanistic gaps, breaking through the bottlenecks of delivery technologies, and accelerating the translation from basic research to clinical applications, so as to provide new strategies for stroke treatment.

**Nomenclature**
BDNF: brain‐derived neurotrophic factor
BMEC: brain microvascular endothelial‐like cells
CIS: cerebral ischemic stroke
DAMP: damage‐associated molecular patterns
ENI: early neurological improvement
ESO: European Stroke Organisation
MCAO: middle cerebral artery occlusion
MSC: mesenchymal stem cells
NeuN: neuronal nuclear
NIHSS: National Institutes of Health Stroke Scale
PAF: platelet‐activating factor
PAMP: pathogen‐associated molecular patterns
RAGE: receptor for advanced glycation end
ROS: reactive oxygen species
SVZ: subventricular zone
TCM: traditional Chinese medicine
TOS: total oxidative status
VEGF: vascular endothelial growth factor

## Author Contributions

Conceptualization: J.Y. and Z.W.; methodology: J.Y. and Z.W.; writing—original draft preparation: J.Y. and Z.W.; software: J.Y., Z.W., T.S., S.W., and P.H.; visualization: T.S., S.W., P.H., D.T., S.X., and J.L.; writing—review and editing: T.S., S.W., P.H., D.T., S.X., J.L., Y.X., and P.P.; supervision: D.T., S.X., and J.L.; J.W. contributed to validation (approving the final version of the manuscript). J.Y., Z.W., and T.S. have contributed equally to this work and share first authorship.

## Funding

This study was supported by National Natural Science Foundation of China (10.13039/501100001809; 82560932); Yunnan Provincial Department of Science and Technology‐Basic Research Program (202201AU070176, 202301AZ070001‐026); the Major Project of University‐Hospital Joint Fund of Yunnan University of Chinese Medicine (XYLH2024001); the Applied Basic Research General Project of Yunnan Province (202201AT070214); the Yunnan Two Talents Program (202205AD160024); the Joint Special Fund for Applied Basic Research of Traditional Chinese Medicine of Yunnan Provincial Science and Technology Department (202001AZ070001‐020, 202301AZ070001‐016); the Scientific Research Foundation of Yunnan Provincial Department of Education (2025Y0618, 2025Y0632, 2024Y391); and the Yunnan Provincial Innovation Team for Prevention and Treatment of Brain Diseases (ZTNB404, ZTNB212).

## Disclosure

All authors have read and agreed to the published version of the manuscript. The statements, opinions, and data contained in all publications are solely those of the individual authors and contributors and not of MDPI and/or the editors. MDPI and/or the editors disclaim responsibility for any injury to people or property resulting from any ideas, methods, instructions, or products referred to in the content.

## Consent

The authors have nothing to report.

## Conflicts of Interest

The authors declare no conflicts of interest.

## Data Availability

The data that support the findings of this study are available from the corresponding authors upon reasonable request.
